# Involvement of the auxin–cytokinin homeostasis in adventitious root formation of rose cuttings as affected by their nodal position in the stock plant

**DOI:** 10.1007/s00425-021-03709-x

**Published:** 2021-09-06

**Authors:** Millicent A. Otiende, Klaus Fricke, Julius O. Nyabundi, Kamau Ngamau, Mohammad R. Hajirezaei, Uwe Druege

**Affiliations:** 1 University of Kabianga, P.O. Box 2030-20200, Kericho, Kenya; 2 Leibniz Institute of Vegetable and Ornamental Crops (IGZ), 99090 Erfurt, Germany; 3 Maseno University, P.O. Box Private Bag, Maseno, Kenya; 4 Jomo Kenyatta University of Agriculture and Technology, P.O. Box 62, Nairobi, 000-00200 Kenya; 5 Leibniz Institute of Plant Genetics and Crop Plant Research, Gatersleben, 06466 Stadt Seeland, Germany; 6 Present Address: Erfurt Research Centre for Horticultural Crops (FGK), University of Applied Sciences Erfurt, 99090 Erfurt, Germany

**Keywords:** Age, Axillary bud growth, Maturation, Plant hormones, Root development, Topophysis

## Abstract

**Main conclusion:**

Enhanced levels of indole-3-acetic and raised auxin to cytokinin ratios in the stem base contribute to the positive acropetal gradient in rooting capacity of leafy single-node stem cuttings of rose.

**Abstract:**

Cuttings excised from different nodal positions in stock plants can differ in subsequent adventitious root formation. We investigated the involvement of the auxin–cytokinin balance in position-affected rooting of *Rosa hybrida.* Leafy single-node stem cuttings of two rose cultivars were excised from top versus bottom positions. Concentrations of IAA and cytokinins were monitored in the bud region and the stem base during 8 days after planting using chromatography–MS/MS technology. The effects of nodal position and external supply of indole-butyric acid on rooting were analyzed. Most cytokinins increased particularly in the bud region and peaked at day two before the bud break was recorded. IAA increased in both tissues between day one and day eight. Top versus bottom cuttings revealed higher levels of isopentenyladenosine (IPR) in both tissues as well as higher concentrations of IAA and a higher ratio of IAA to cytokinins particularly in the stem base. The dynamic of hormones and correlation analysis indicated that the higher IPR contributed to the enhanced IAA in the bud region which served as auxin source for the auxin homeostasis in the stem base, where IAA determined the auxin–cytokinin balance. Bottom versus top cuttings produced lower numbers and lengths of roots, whereas this deficit was counterbalanced by auxin application. Further considering other studies of rose, it is concluded that cytokinin-, sucrose- and zinc-dependent auxin biosynthesis in the outgrowing buds is an important factor that contributes to the enhanced IAA levels and auxin/cytokinin ratios in the stem base of apical cuttings, promoting root induction.

**Supplementary Information:**

The online version contains supplementary material available at 10.1007/s00425-021-03709-x.

## Introduction

Adventitious root (AR) formation is a fundamental biological process by which new roots are formed post-embryonically from cells of non-root tissues (Steffens and Rasmussen 2016). Excision-induced AR formation in the stem base of shoot-borne cuttings, following their removal from a donor plant, is the most important basis for clonal propagation of horticultural and forestry plants. AR formation involves successive developmental phases (da Costa et al. 2013; Druege et al. 2019). The initial phase, mostly referred to as the induction phase, is characterized as an anatomical lag phase, during which the initial cell reprogramming occurs. If the cells from which AR starts (AR source cells) are root-competent already, they can be fate-converted directly to AR root founder cells by a root-inducing signal. However, often the cells first have to acquire root competence involving dedifferentiation. After determination of AR founder cells, new roots are formed by cell division, differentiation and growth. It is widely accepted that auxin, mainly indole-3-acetic acid (IAA), is the central player that induces ARs in competent cells. However, auxin action is cross-linked to other plant hormones, while the endogenous control is still under-explored (da Costa et al. 2013; Lakehal and Bellini 2019; Druege et al. 2019). Among the co-players, cytokinins (CKs) are thought to have an early positive regulative function during dedifferentiation, whereas high CK levels act antagonistic to auxin during the induction phase (da Costa et al. 2013; Druege et al. 2019).

Formation of ARs in cuttings is highly dependent on diverse environmental factors at stock plant and cutting levels, the ontogenetic stage of the stock plant and the position of cuttings within the stock plant (Druege 2020). For positional effects the term ‘topophysis’ has been repeatedly used. It has originally been introduced as ‘Ortsnatur’ by Molisch (1916) describing the potential of a cutting for subsequent growth and differentiation depending on its spatial origin within the donor plant. Effects of the cutting position within the shoot on subsequent rooting is a general phenomenon that has been described for nodal cuttings of several plant species such as *Hedera helix* (Poulsen and Andersen 1980), *Schefflera arboricola* (Hansen 1986), *Stephanotis floribunda* (Hansen 1989), *Rosa multiflora* (Hambrick et al. 1991) and *Corymbia torelliana* × *C. citriodora* (Wendling et al. 2015). The mechanisms underlying such effects of nodal position are not known.

Position effects along the shoot on rooting capacity of cuttings can principally result from three types of influences, environment, age and relative position within the stock plant. For example, in vertically grown shoots, lower positions may decrease the effective light intensity and red/far-red ratio due to shading by the upper part (Iglesias et al. 2018). This may result in lower carbohydrate levels or modified auxin homeostasis and signaling in the cuttings, while both factors affect rooting (Rapaka et al. 2005; Ruedell et al. 2015). Cuttings excised from upper shoot positions have a different age compared to lower position cuttings. They are chronologically younger based on the shorter time since the tissue differentiated from the shoot apical meristem (Rasmussen et al. 2015). This may involve less lignification and sclerenchyma development, which has been correlated to the better rooting of top position eucalypt (*C. torelliana* × *C. citriodora*) cuttings (Wendling et al. 2015). At the same time, tissues from top positions have undergone more cell divisions since the meristem has been laid down so that they have a less juvenile but more mature character than bottom positions of the shoots (Poethig 1990; Rasmussen et al. 2015). The underlying “vegetative phase change” from juvenile to adult character of the tissues during shoot growth is gradual (Poethig 1990, 2013). Two microRNAs, miR156 and miR157, and their direct targets, the SPL (SQUAMOSA PROMOTER BINDING PROTEIN-LIKE) family of transcription factors have important regulative functions during vegetative phase transition, while the regulatory network is further linked to sugars and plant hormones (Poethig 2013). A maturation-induced decline in rooting capacity of cuttings has been frequently observed with forest tree species and changes in auxin homeostasis and signaling seem to be involved (Pizzaro and Díaz-Sala 2019). At a given environment and age of the excised cutting, its physiological condition is further affected by the relative position within the whole plant network, which means its proximity to sources and sinks and its consequential supply status with resources such as mineral nutrients and carbohydrates and systemic signals such as plant hormones that function during AR formation.

Among the plant hormones, auxins and CK concentrations can particularly be expected to vary along the shoot of a stock plant. In shoots, the most important physiologically active auxin IAA is synthesized in young expanding leaves and buds and is in the stem either transported root-ward by polar auxin transport (PAT) in xylem parenchyma and cambium cells or co-transported in the phloem associated with assimilate transport (Kramer and Bennett 2006; Petrasek and Friml 2009; Leyser 2011; Barbier et al. 2015). Both pathways are supplemented with a low conductance and less polar “connective auxin transport”, linking the PAT route to the surrounding tissues, and can be interconnected in leaves (Cambridge and Morris 1996; Bennett et al. 2016; van Rongen et al. 2019). Among the CKs, the free-base CKs such as *trans*-zeatin (tZ) and isopentenyladenine (IP) are regarded as physiologically active, whereas their sugar conjugates (nucleosides) and derived phosphate conjugates (nucleotides) are considered as inactive transport and storage forms (Davies 2010; Sakakibara 2010). After the traditional view that CKs are exclusively synthesized in the root and transported to the shoot through the xylem, there has been provided evidence, that CKs can be synthesized also in various aerial sites such as leaves, stems and flowers while long-distance distribution involves both the xylem and phloem route. In this context, *trans*-zeatin riboside (tZR) was shown to be transported acropetally in xylem sap whereas isopentenyladenosine (IPR) was transported basipetally via phloem, respectively (Kudo et al. 2010; Skalicky et al. 2018).

**Fig. 1:**
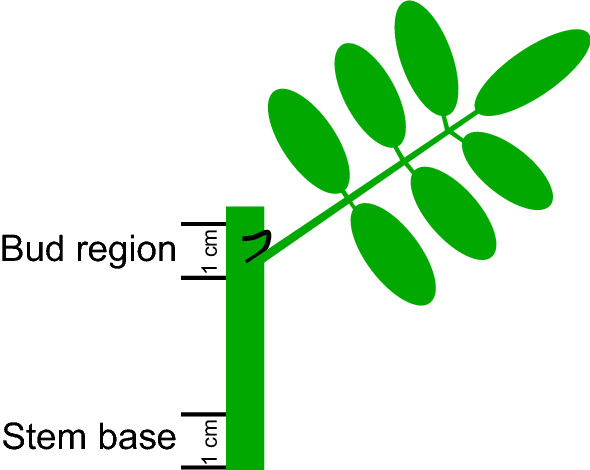
Leafy stem cuttings of *Rosa hybrida* (circa 4–5 cm in length) and the two stem sections sampled for plant hormone analysis. The stem section of the bud region was sampled with the bud but without leaves

*Rosa hybrida* is increasingly propagated by rooting of leafy nodal cuttings, consisting of a stem, approximately 5 cm in length, one 5–7-leaflet leaf and one axillary bud (Fig. 1). Studies of such rose cuttings support an acropetal increase of rooting capacity along the shoot. Comparing the most apical with the lower six node positions of the cultivars ‘Korokis’, ‘Tanettahn’ and ‘Sweet Promise’, the bottom position cuttings revealed the lowest percentage of rooted cuttings (Bredmose and Hansen 1996). Accordingly, apical cuttings of the cultivars ‘Pink Song’, ‘Orange Beauty’, ‘Gummack’ showed the highest rooting percentage (Park et al. 2011). Otiende et al. (2017) studied position effects on rooting of *Rosa hybrida* ‘Natal briar’ and ‘Rosa progress’ and found, that number, length and dry mass of ARs of increased along the bottom, middle and top position of vertical, 1.5 m long shoots.

Considering these relationships, the present study aimed to elucidate the influence of nodal position of rose cuttings on auxin and CK homeostasis and its potential role in position-affected AR formation. Based on the findings of Otiende et al. (2017), we investigated whether the two cultivars ‘Natal Briar’ and ‘Rosa Progress’ can be characterized by a similar acropetal shift of auxin–cytokinin patterns during the early rooting period. Top and bottom cuttings of both cultivars were planted and concentrations of IAA and diverse CKs were monitored in the stem base as the zone of AR regeneration and in the bud region as the zone of bud outgrowth. Following the results, we analyzed the response of adventitious root formation of top versus bottom cuttings to basal application of increasing auxin concentrations.

## Materials and methods

### Plant material

Cuttings of the *R. hybrida* cultivars ‘Natal Briar’ and ‘Rosa progress’ were used. ‘Natal briar’ has thorns and been described as easy to root (de Vries 2003). ‘Rosa progress’ is a thornless cultivar that in preliminary studies revealed similar rooting characteristics as ‘Natal Briar’, while cuttings of both cultivars showed an acropetal increase in rooting capacity (Otiende et al. 2017). Cuttings of both cultivars were harvested from 3-month-old stock plants maintained in the greenhouse. Vertical shoots measuring ca. 135 cm in length were cut from the plants. From each shoot, two nodal cuttings, each bearing one bud in the axil of a 5-leaflet leaf (Fig. 1) were excised out of the centers of the apical third (top position) and the basal third (bottom position).

### Rooting conditions

Each cutting was planted in a pot (7 cm x 7 cm × 6.5 cm) filled with clean sterile coccus [SL (PVT), Sri Lanka, supplied by Hardi Kenya, Nairobi, Kenya] with a pH of 6.5–7.5 and electrical conductivity of 0.18–0.24 mS cm^−1^. The following conditions were maintained in the greenhouse: temperatures of 30–35 °C at day time and 22–24 °C at night, relative humidity of ≥ 90% provided by misting cycles of 10–30 min at day time and 1–2 h at night. After 2 weeks, the misting was gradually reduced. A thermal screen was applied to control the light intensity. The average light intensity in the greenhouse during the day was 630 µmol m^−2^ s^−1^ (PPFD). Fertigation started at 14 days after planting (DAP) and every 4 days thereafter aiming at an EC of 1.3–1.5 mS cm^−1^. The fertilizers used were Ca(NO_3_)_2_, KNO_3_, (NH_4_)_2_SO_4_, urea, MgSO_4_, iron chelates, MnSO_4_, ZnSO_4_, BNa_3_O_3_, Na_2_MoO_4_ and CuSO_4_ from Amiran, Nairobi, Kenya (Otiende et al. 2017).

### Monitoring of plant hormone levels

The experiment was laid out in a randomized complete block design including four replications. The treatments consisted of two cutting positions (top and bottom) in a factorial combination with two rootstock cultivars (‘Natal briar’ and ‘Rosa progress’). Per each genotype, four lots of top cuttings and four lots of bottom cuttings, each consisting of 25 cuttings (in total 100 cuttings per cultivar and cutting position) were harvested as described. From each lot, five cuttings were sampled to analyze the hormone levels at planting date (0 DAP) and the other 20 cuttings were planted and cultivated as described for the analysis of hormone levels at 1, 2, 4 and 8 DAP. At each day, between 10.00 and 10.30 a.m. samples from the stem base and bud region (Fig. 1) were collected from five cuttings per replicate, pooled per tissue, shock frozen under liquid nitrogen and stored in a freezer at − 80 °C.

### Extraction and analysis of plant hormones

The frozen samples were ground in a micromill (Retsch MM301, Haan, Germany), together with five stainless steel balls (diameter 3 mm) for 5 min at a vibration frequency of 30 vibrations s^−1^.

Auxin analysis focused on IAA as most important physiologically active and actively transported auxin that is essential for induction of ARs (Lakehal and Bellini 2019). The extraction and analysis of IAA by GC–MS/MS is described in detail by Ahkami et al. (2013). From the homogenized frozen plant material, 150 mg of fresh weight were transferred into a 1.5 ml Eppendorf vial. IAA was extracted with methanol (20 min at 60 °C, on a shaker) supplemented with 2.6 pmol (^2^H)_2_-IAA as internal standard. After centrifugation at 14,000*g*, the supernatant was cleaned-up by a solid-phase extraction procedure described by Ahkami et al. (2013) using aminopropyl solid-phase extraction columns (Chromabond NH_2_ shorty 10 mg; Machery-Nagel, Dueren, Germany). Samples were methylated using ethereal diazomethane. Separation and mass fragment analysis were conducted using a Varian Saturn 2200 ion-trap mass spectrometer connected to a CP-3800 gas chromatograph (Agilent, Santa Clara, CA, USA) fitted with a CombiPal autoinjector (CTC Analytics AG, Zwingen, Switzerland) as described in detail by Ahkami et al. (2013). Settings for endogenous IAA were chosen as follows: parent ion (*m/z*) = 190 (M+H)^+^, diagnostic product ion (*m/z*) = 130. A second channel analyzing the isotopically labeled standard (2H)^2^-IAA used the parent ion (*m/z*) = 192 (M+H)^+^ and the diagnostic daughter ion (*m/z*) = 132. The amount of endogenous compound was calculated from the signal ratio of the unlabeled over the corresponding stable isotope-containing mass fragments.

Extraction and analysis of CKs followed the protocol of Rasmussen et al. (2015). One ml of ice-cold solution methanol:formic acid:water in a ratio of 15:1:4 (by vol.) was added to 100–140 mg of frozen homogenized sample. The homogenate was thoroughly mixed and stored at − 20 °C for 16 h. After incubation, the homogenate was centrifuged at 4 °C for 20 min at 13,000*g*. The supernatant was transferred to a 2 ml tube and the remaining pellet was re-extracted with 300 µl of extraction buffer and centrifuged. The combined supernatants were reduced to dryness in a vacuum centrifuge at 40 °C for 3½ h at 200 mbar. To prepare the clean-up by solid-phase extraction, the dried samples were re-suspended in 100 µl 80% methanol, followed by the addition of 900 µl of 1 M formic acid. Separation of the different hormones was done using MCX columns (Oasis^®^ MCX Sorbent). These were equilibrated by successive addition of 1 ml of acetonitrile, 1 ml of methanol, 1 ml of 1 M formic acid, and finally, 1 ml of 0.1 M HCl. After the sample was loaded onto the column, 1 ml of 1 M formic acid and 1 ml pure MeOH were successively added to remove the acidic and neutral hormones. Then, the CKs were eluted with 1 ml 0.35 M ammonia (NH_3_) dissolved in 60% MeOH. Eluents were dried under vacuum at 38 °C for about 3 ½ h and then re-dissolved in 50–100 µl of 25% MeOH.

Analysis of CKs considered the physiologically active free bases of the zeatin- and isopentenyl-type and their nucleosides that have important functions in cytokinin transport. It was conducted essentially as described by Mayta et al. (2018). Briefly, an Agilent 1290 Infinity system connected to an Agilent triple quadrupole mass spectrometer QQQ6490 was used for separation and detection of individual hormones. Separated compounds were ionized at atmospheric pressure via electrospray and directed to the mass spectrometer. The control of the complete system and recording of the spectra were performed with the MassHunter, release B.04.00 (B4038). The following parameters were employed: desolvation temperature 350 °C, desolvation nitrogen gas of 720 l h^−1^ for both, capillary voltage 2.0 kV, detection in positive ion mode and different dwell times between 20 and 200 s. Collision energy settings for the compounds are given in (Rasmussen et al. 2015). Multiple reactions monitoring (MRM) was performed to identify individual compounds accurately. The calculation of the identified CKs was done based on the authentic pure external standards with different concentrations for each compound. Levels of tZR and of IPR were further corrected by the recovery of their labeled forms that were added to each sample during the extraction. For this purpose and for testing the stability of the instruments, a mixture of [2H5]–tZR and [2H6]–IPR was used. Separation of hormones was performed on an Eclipse Plus C18 column, RPHD 1.8 mm, 2.1 × 50 mm. The gradient was accomplished with 0.1% (v/v) formic acid in LCMS grade water as buffer A and 0.1% (v/v) formic acid in LCMS grade methanol (Chemsolute) as buffer B. The column was equilibrated with a mixture of buffer A (86.5%) and B (13.5%) at a flow rate of 0.4 ml min^−1^ and heated at 40 °C during the whole measurement. The gradient was produced by changes of buffer B as follows: 0–5 min at 18%, 5–6 min at 70%, 6–7 min at 99%, 7–7.1 min at 13.5%, and kept up to 9 min at 13.5%. The whole duration of the run was 9.0 min.

### Analysis of rooting and its response to extern auxin application

Number and length of produced adventitious roots were determined at the level of each cultivar as affected by nodal position and external auxin supply. At planting, the basal ends of the cuttings either remained untreated or were dipped in powdered form of indole-3-butyric acid (IBA) (w/w-indole-3-butyric acid, Rhizopon, Rhizopon BV) at concentrations of 0.2%, 0.4% and 0.6%. Each treatment combination was replicated three times, while each replication consisted of 20 cuttings.

Number and length of ARs were evaluated at day 30 after planting. Primary roots per cutting were counted and length of primary roots was measured using a ruler.

### Statistical analysis

Mean values and standard errors (SE) were calculated. Data was analyzed using Statistica Version 3, TIBCO Software Inc. (Palo Alto, CA, USA). The data were subjected to two-factor or three-factor analysis of variance (ANOVA). In case of significant interactions, mean values were compared using the Tukey test (*P* ≤ 0.05). Linear regressions and coefficients of determination were determined to analyze interrelationships between specific hormone data.

## Results

**Fig. 2:**
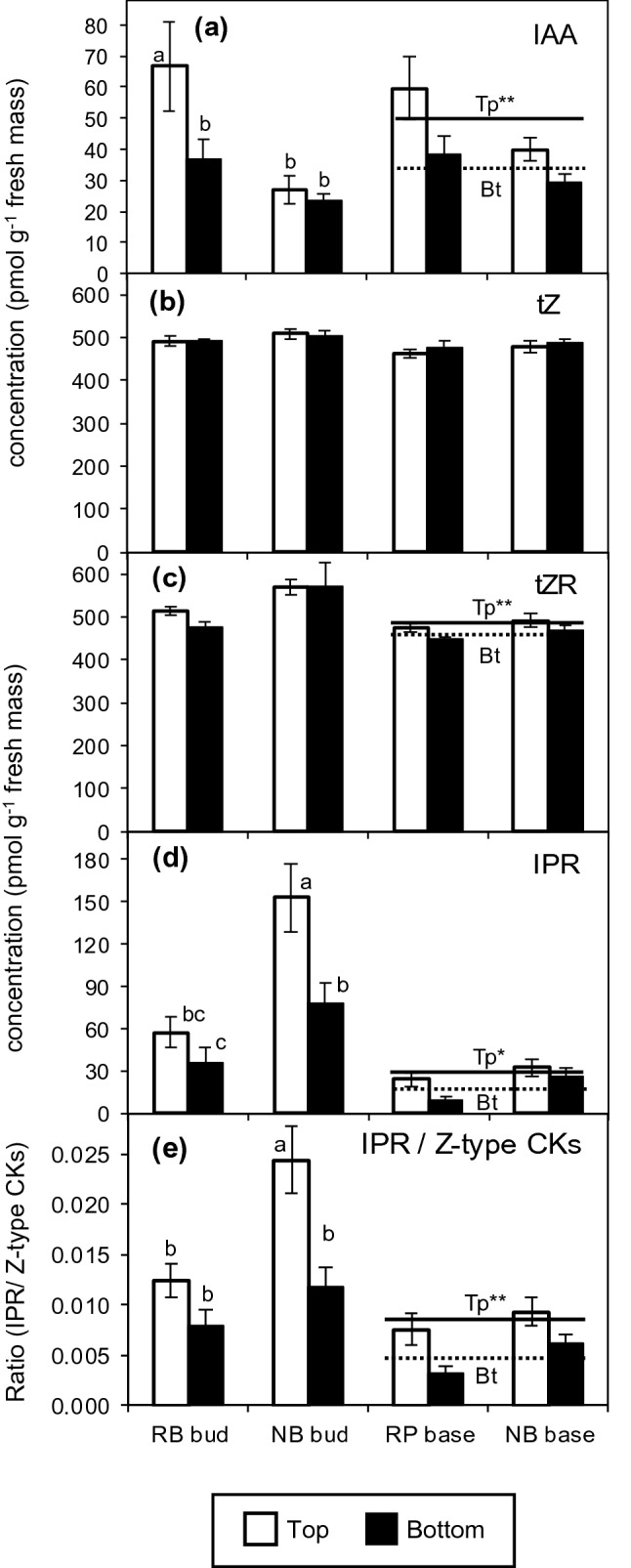
Effect of cutting position on the mean concentrations calculated over the period from 0 to 8 days after planting **a** indole-3-acetic acid (IAA), **b** *trans*-zeatin (tZ), **c** *trans*-zeatin riboside (tZR), **d** isopentenyladenosine (IPR), and **e** the mean ratio of IPR to Z-type CKs in the bud region and stem base of rose cuttings of the cultivars ‘Rosa progress’ (RP) and ‘Natal Briar’ (NB). Columns and bars represent the mean values and SE at cultivar level (*n* = 20, each consisting of tissue from five cuttings). When the effect of cutting position was significant at *P* ≤ 0.05 (*) or P ≤ 0.01 (**) and independent on cultivar, continuous and broken lines represent the mean levels of both cultivars for top (Tp) and bottom (Bt) cuttings, respectively. In cases of a significant interaction between cutting position and cultivar, columns that do not share a common letter, represent significantly different levels (Tukey test, *P* ≤ 0.05)

**Fig. 3:**
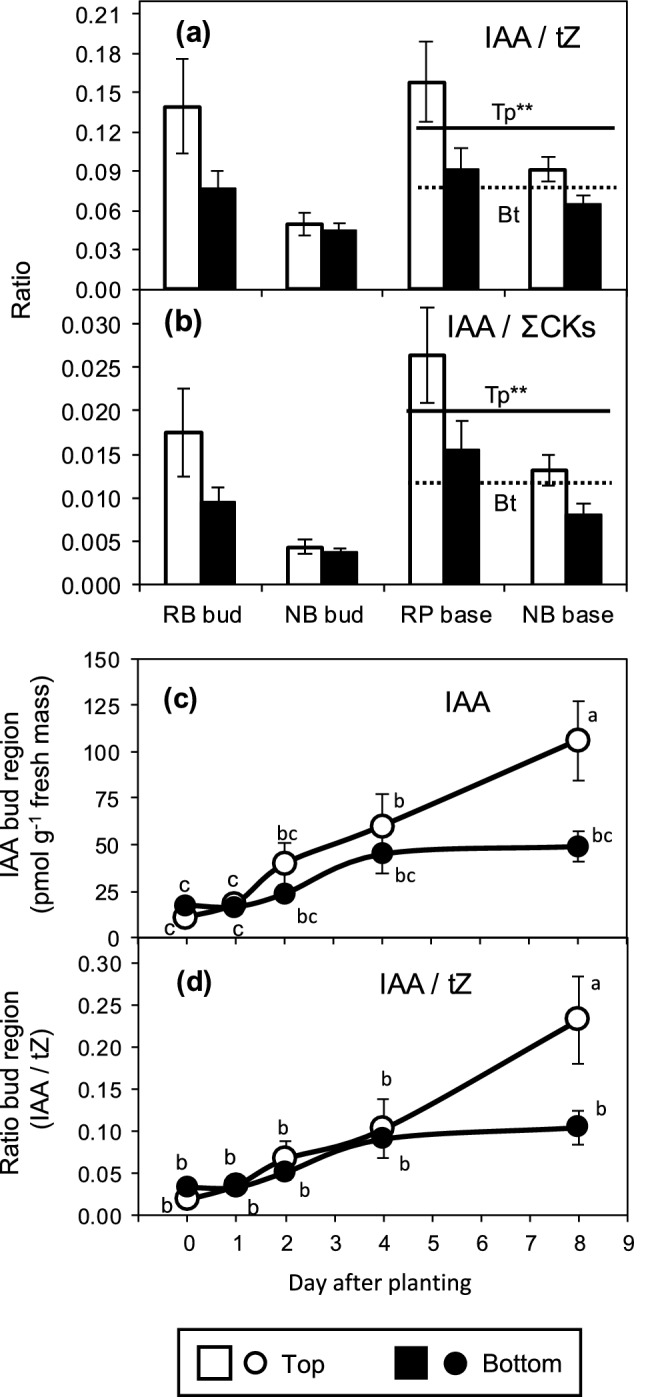
Effect of cutting position on the mean ratios calculated over the period from 0 until 8 days after planting between **a** indole-3-acetic acid (IAA) and *trans*-zeatin (tZ), and **b** IAA and total cytokinins (∑CKs) in the bud region and stem base of rose cuttings of the cultivars ‘Rosa progress’ (RP) and ‘Natal Briar’ (NB). Columns and bars represent the mean values and SE at cultivar level (*n* = 20, each consisting of tissue from five cuttings). Continuous and broken lines represent the mean levels of both cultivars for Top (Tp) and Bottom (Bt) cuttings, respectively. **c** Mean concentrations of IAA and **d** mean ratios of IAA to tZ of both cultivars as affected by the interaction between cutting position and time after planting. **Main effect of cutting position significant at *P* ≤ 0.01 and independent on cultivar. Symbols that do not share a common letter, represent significantly different levels (Tukey test, *P* ≤ 0.05)

**Fig. 4:**
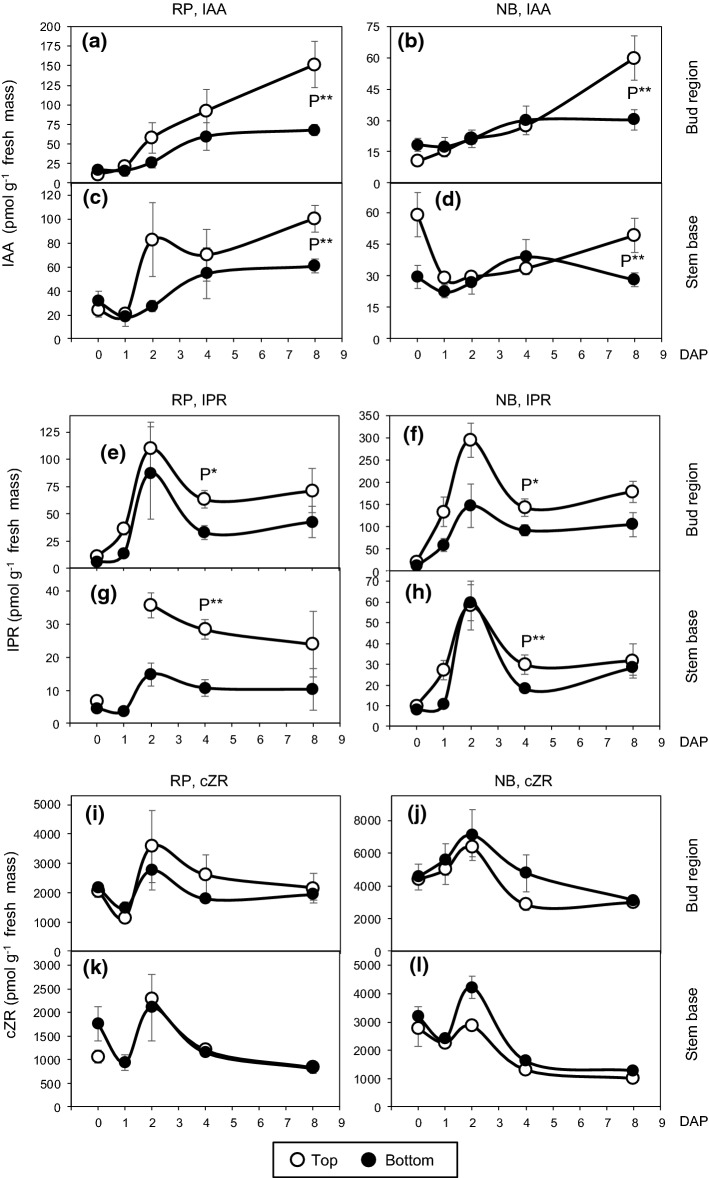
Temporal distribution of indole-3-acetic acid (IAA, **a–d**), isopentenyladenosine (IPR, **e–h**) and *cis*-zeatin riboside (cZR, **i–l)** in the bud region (**a, b, e, f, i, j**) and the stem base (**c, d, g, h, k, l**) of rose cuttings of the cultivars ‘Rosa progress’ (RP) and ‘Natal briar’ (NB) as affected by the cutting position (top, bottom). Mean values ± SE (*n* = 4, each consisting of tissue from five cuttings). Curve fit of CK levels in the stem base of top RP cuttings interrupted because no data exist for 1 DAP. P*, P** significant effect of cutting position independent on cultivar at the specific DAP at *P* ≤ 0.05, 0.01, respectively

**Fig. 5:**
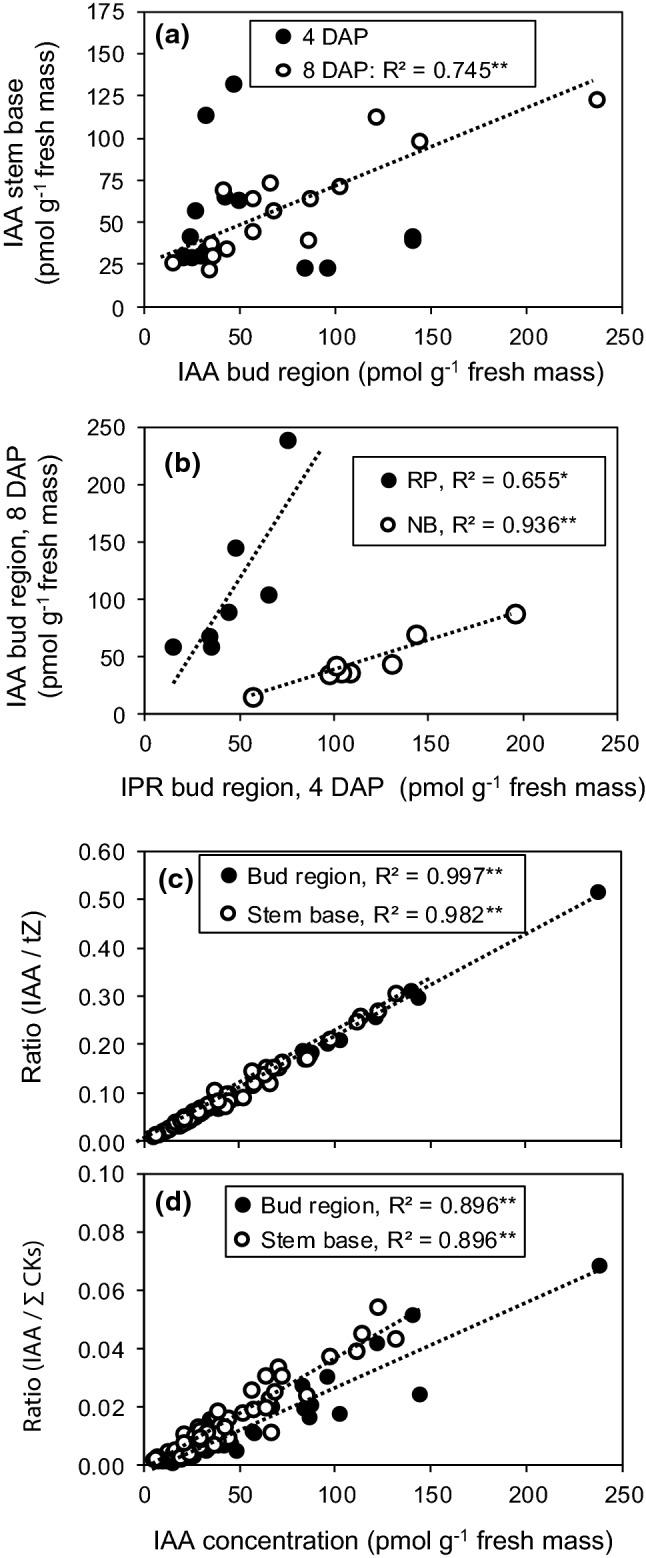
Scatterplots of **a** IAA concentration in the bud region (*x*) and IAA concentration in the stem base (*y*), **b** IPR concentration in the bud region at 4 DAP (*x*) and IAA concentration in the same tissue at 8 DAP (*y*), **c, d** IAA concentrations in the bud region or stem base at 4 DAP and 8 DAP (*x*) and the ratios of **c** IAA to tZ or **d** IAA to the sum of cytokinins (∑CKs) in the same tissue (*y*). Pooled data of both cultivars and both cutting positions in **a, c, d** and data per cultivar in **b**. *n* = 16 for each DAP (**a**), *n* = 7 for ‘Rosa progress’ (RP) and *n* = 8 for ‘Natal briar’ (NB) (**b**), *n* = 73 for the bud region, *n* = 64 for the stem base (**c**, **d**). Each *n* consisting of tissue from five cuttings. *, **Significant *R*^2^ at *P* ≤ 0.01, 0.05, respectively. Dotted lines illustrate the linear trends

**Fig. 6:**
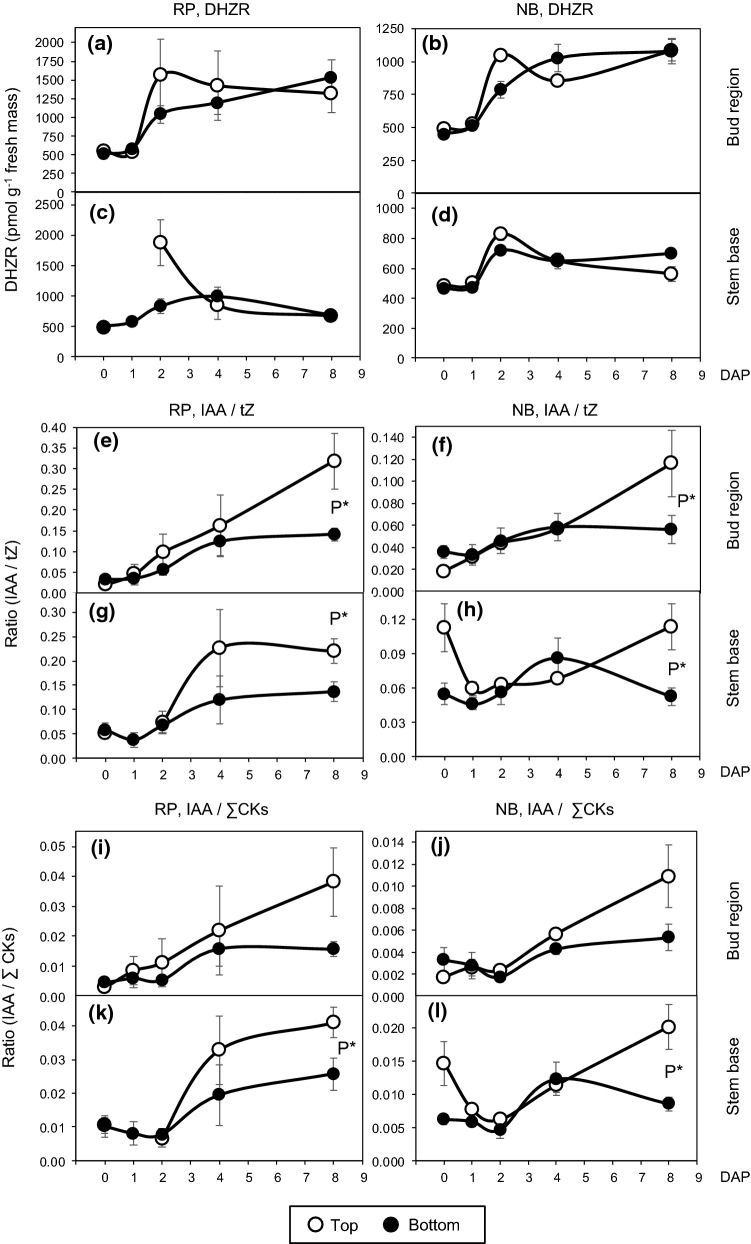
Temporal distribution of dihydrozeatin riboside (DHZR, **a–d**), the ratio of indole-3 acetic acid (IAA) to *trans*-zeatin (tZ) (**e–h**) and the ratio of IAA to total cytokinins (∑CKs) (**i–l**) in the bud region (**a, b, e, f, i, j**) and the stem base (**c, d, g, h, k, l**) of rose cuttings of the cultivars ‘Rosa progress’ (RP) and ‘Natal briar’ (NB) as affected by cutting position (top, bottom). Mean values ± SE (*n* = 4, each consisting of tissue from five cuttings). Curve fit of ratios in the stem base of RP top cuttings interrupted because no data exist for 1 DAP. P*, significant effect of cutting position independent on cultivar at the specific DAP at *P* ≤ 0.05

We investigated the involvement of the auxin–cytokinin balance in position-affected rooting of *Rosa hybrida*, regarding potential interferences with plant genotype. To this end, we analyzed the influence of two nodal positions of cuttings (top versus bottom) within the shoots of the stock plant on the temporal distribution of IAA and of zeatin- and isopentenyl-type CKs in the bud region and the stem base, comparing the two cultivars ‘Natal Briar’ and ‘Rosa Progress’. The ANOVA results of the effects of cultivar, cutting position and time after planting on the hormone levels in the two cutting sections are summarized in Table 1. According to Table 1, the hormone levels and ratios as affected by cutting position and its interaction with cultivar and time after planting are illustrated in Figs. 2 and 3. In addition, Figs. 4 and 6 provide coherent pictures of the dynamic of the plant hormones and ratios in the bud region and the stem base per each cultivar as affected by the cutting position. Figure 5 illustrates relationships between specific hormones and ratios, calculated for specific tissues and dates.

**Table 1:** ANOVA results of the effects of cultivar (C), cutting position (P), and time after planting (T) on concentrations of plant hormones in the bud region (a) and the stem base (b) of rose cuttings (*n* = 4)

Hormone	C	P	T	C × P	C × T	P × T	C × P × T
(a) Bud region							
IAA	**	**	**	*	**	**	ns
tZ	ns	ns	**	ns	ns	ns	ns
tZR	ns	ns	ns	ns	ns	ns	ns
cZR	**	ns	*	ns	ns	ns	ns
DHZR	**	ns	**	ns	ns	ns	ns
IPR	**	**	**	*	*	ns	ns
Total CKs	**	ns	ns	ns	ns	ns	ns
IPR/(Z-type CKs)	**	**	**	*	*	ns	ns
IAA/tZ	**	*	**	ns	**	*	ns
IAA/total CKs	**	ns	**	ns	ns	ns	ns
(b) Stem base							
IAA	**	**	**	ns	**	ns	ns
tZ^R^	ns	ns	**	ns	ns	ns	ns
tZR^R^	*	**	**	ns	ns	ns	ns
cZR^R^	**	ns	**	ns	*	ns	ns
DHZR^R^	**	*	**	*	**	**	**
IPR^R^	**	*	**	ns	*	ns	ns
Total CKs^R^	**	ns	**	*	**	ns	**
IPR/(Z-type CKs)^R^	**	**	**	ns	ns	ns	ns
IAA/tZ^R^	**	**	**	ns	**	ns	ns
IAA/total CKs^R^	**	**	**	ns	**	ns	ns

*IAA* indole-3-acetic acid, *IPR* isopentenyladenosine, *tZ* *trans*-zeatin, *tZR* *trans*-zeatin riboside, *cZR* *cis*-zeatin riboside, *DHZR* dihydrozeatin riboside

*, **Significant at *P* ≤ 0.05, 0.01, respectively; ns, non-significant. Z-type CKs represent the sum of the cytokinins (CKs) tZ, tZR, cZR, DHZR

^R^Excluding day 1 because of destroyed samples of top cuttings of cultivar ‘Rosa progress’

### Auxin concentrations in cutting tissues

IAA concentrations in the bud region were affected by cultivar, cutting position, the time after planting and the two-way interactions among these factors (Table 1). As a result, the average IAA levels in the bud region, calculated over the 8-day-period, were higher in top than in bottom cuttings for ‘Rosa progress’ but not for ‘Natal briar’ (Fig. 2a). IAA in the stem base was also affected by each single factor but the effect of cutting position was not subject to interaction with any other factor (Table 1). Consequently, mean IAA levels in the stem base over the 8-day-period were generally higher in top than in bottom cuttings for both cultivars (Fig. 2a). The temporal analysis of IAA in the bud region revealed a slight increase between day 1 and day 4 after planting (DAP) in both cutting types (Fig. 3c). Whereas IAA concentrations in the bud region of bottom cuttings remained on a same level thereafter, IAA in top cuttings strongly increased until 8 DAP to reach significantly higher levels compared to bottom cuttings (Fig. 3c). Considering the temporal course of IAA levels at cultivar level, IAA in the stem base of both cultivars showed a transient decrease until 1 DAP and increased thereafter (Fig. 4c, d). The statistical analysis did not reveal a significant interaction between cutting position and time on the IAA levels in the stem base (Table 1). However, when considering the individual time points at cultivar level (Fig. 4c, d), a consistent effect of cutting position was found between 4 and 8 DAP. During this period, the IAA concentrations in the stem base increased only in top cuttings of both cultivars and at 8 DAP reached significantly higher levels than the bottom cuttings, which corresponded to the similar increase of IAA in the bud region (Fig. 4a–d). These results suggested a common principle for both cultivars that underlies the acropetal rise of IAA levels in the stem base and indicated a linkage to the bud region.

In addition to local processes controlling auxin homeostasis, auxin accumulation in the stem base of cuttings may depend on auxin influx from upper cutting parts (Garrido et al. 2002; Yang et al. 2019). The correspondence of the IAA dynamic between the bud region and the stem base and the finding, that the acropetal gradient in IAA levels at 8 DAP was greater in the bud region compared to the stem base region (Fig. 4a–d), may indicate an auxin source function of the bud region for the enhanced IAA accumulation in stem base of top cuttings. To find more statistical support for the linkage of IAA between the two cutting regions, the correlation between IAA levels in the bud region as independent variable and IAA levels in the stem base as dependent variable was analyzed at the level of individual replicates. When the data pool of 0, 1 and 2 DAP of both cultivars was analyzed, IAA concentrations in the stem base were not related to the IAA concentration in the bud region (coefficient of determination *R*^2^ = 0.030, n.s., *n* = 47). This also applied to the IAA data of 4 DAP (*R*^2^ = 0.017, *n* = 16, Fig. 5a). However, the IAA concentration in the stem base at 8 DAP showed a strong positive relationship to the simultaneous IAA concentration in the bud region, which explained almost 75% of its variability (Fig. 5a).

### Cytokinin concentrations in cutting tissues

Using the LC–MS/MS protocol described, tZ, tZR, *cis*-zeatin riboside (cZR), dihydrozeatin riboside (DHZR) and IPR were detected in the rose cutting tissues. We could not detect IP and dihydrozeatin. In the bud region, only IPR was affected by cutting position, while this effect was further dependent on cultivar (Table 1, Fig. 2d). In the stem base, IPR and also tZR were affected by cutting position independent on cultivar (Table 1, Fig. 2c, d). It becomes apparent from the right panel in Fig. 2c that even though this position effect on tZR was significant, top position cuttings had only marginally higher tZR levels compared to bottom cuttings.

Most cytokinins such as IPR (Fig. 4e–h), cZR (Fig. 4i–l), and DHZR (Fig. 6a–d) increased in both tissues after planting and peaked at 2 DAP, the time before the first bud break was recorded (Table 2). The time course of IPR at cultivar level highlights that top cuttings almost continuously contained higher IPR levels than bottom cuttings in both tissues, while the levels were more than twofold higher in the bud region compared to the stem base (Fig. 4e–h). The most consistent effect of cutting position on IPR was found at 4 DAP. At this time point, IPR levels in the bud region and in the stem base were higher in top cuttings than in bottom cuttings, while this effect was independent on cultivar (Fig. 4e–h). Most interestingly, the IPR levels in the bud region at 4 DAP revealed significant positive relations to the IAA level in same tissues determined at 8 DAP (Fig. 5b), suggesting a positive function of IPR in IAA accumulation. DHZR levels in the stem base were also affected by cutting position (Table 1). However, this cytokinin was further subject to all two-way interactions and three-way interaction with cultivar and time after planting (Table 1), so that no consistent effect of cutting position could be found (Fig. 6a–d). Concentrations of cZR were very high compared to tZR (compare Fig. 2c with Fig. 4i–l) but were not affected by cutting position in any of the two tissues, which also applied to the sum of CKs (Table 1, Supplemental Fig. S1).

**Table 2:** Most advanced developmental stages recorded in the bud region and the stem base of rose cuttings at the time of sampling for hormone analysis (*n* = 4)

Time	Bud region				Stem base			
DAP	RP	RP	NB	NB	RP	RP	NB	NB
	Top	Bottom	Top	Bottom	Top	Bottom	Top	Bottom
0	u	u	u	u	u	u	u	u
1	u	u	u	u	u	u	u	u
2	u	u	u	u	u	u	u	u
4	B	B	B	u	u	u	u	u
8	E	E	E	B	C	u	S	S

DAP, days after planting; RP, ‘Rosa progress’; NB, ‘Natal Briar’; u, unchanged; B, bud break (increased size of buds); S, swelling (increased diameter); C, callusing (mass of undifferentiated cells at the wound site); E, shoot emergence (shoot ca. 0.2 cm in length)

Considering the already discussed relationship between position and age of cuttings and findings in other plant species that plant age can affect the ratio of IPR to zeatin-type CKs, we analyzed this parameter. Top cuttings contrasted to the bottom cuttings by a strongly enhanced ratio of IPR to zeatin-type cytokinins in the stem base independent on cultivar, while in the bud region this effect was restricted to ‘Natal Briar’ (Fig. 2e). For the specific combinations of cultivar and cutting position, the temporal courses of the IPR/zeatin-type CKs ratios (Supplemental Fig. S2) were similar to the IPR levels (Fig. 4e–h). Exclusion of cZR from the pool of zeatin-type CKs principally did not affect these results (Supplemental Fig. S3).

### Auxin:cytokinin balance

According to the current understanding, the auxin to cytokinin balance in the tissues of root regeneration influences AR formation, because high-cytokinin levels act antagonistically to auxin during AR induction (Druege et al. 2019). Considering the known physiological activity of tZ but accounting also for potential activities of the other CKs, we calculated the ratios of IAA to both, tZ and to the sum of CKs (total CKs). The IAA/tZ ratios in the bud region and the stem base were affected by cutting position, while in the bud region this effect was further subject to time after planting (Table 1). It becomes apparent from Fig. 3d and Fig. 6e–f, that the IAA/tZ ratio in the bud region increased independently on cultivar until 4 DAP in cuttings of both positions but thereafter increased only in top cuttings, at 8 DAP reaching significantly higher values than the bottom cuttings. In the stem base, the IAA/tZ ratio was higher in top cuttings and not subject to interaction with time or cultivar (Fig. 3a). However, similar to the IAA levels, the most consistent effect of cutting position on the IAA/tZ ratio at cultivar level was found at 8 DAP (Fig. 6g, h). The IAA/total CKs ratio in the bud region did not show a clear response to cutting position but in the stem base was significantly enhanced in top versus bottom cuttings (right panel in Fig. 3b), while this effect was independent on other factors (Table 1). The temporal courses of IAA/total CKs ratio in the different tissues and cultivars (Fig. 6i–l) were similar to those of the IAA/tZ ratio (Fig. 6e–h). Furthermore, temporal and factorial patterns of both ratios resembled to those of the IAA level (Fig. 4a–d). Correlation analysis between the IAA concentration in the two cutting parts as independent factor and the ratios of IAA to tZ and total cytokinins in the same tissues as dependent factors revealed strong positive linear relationships (Fig. 5c, d), while the IAA level explained more than 98% of the variability of the IAA/tZ ratio and almost 90% of the variability of the IAA/total CKs ratio. These findings highlighted IAA as the dominating compound, which affected the auxin–cytokinin balance in the bud and stem base region under the influence of cultivar and cutting position.

### Rooting response to exogenous auxin

**Fig. 7:**
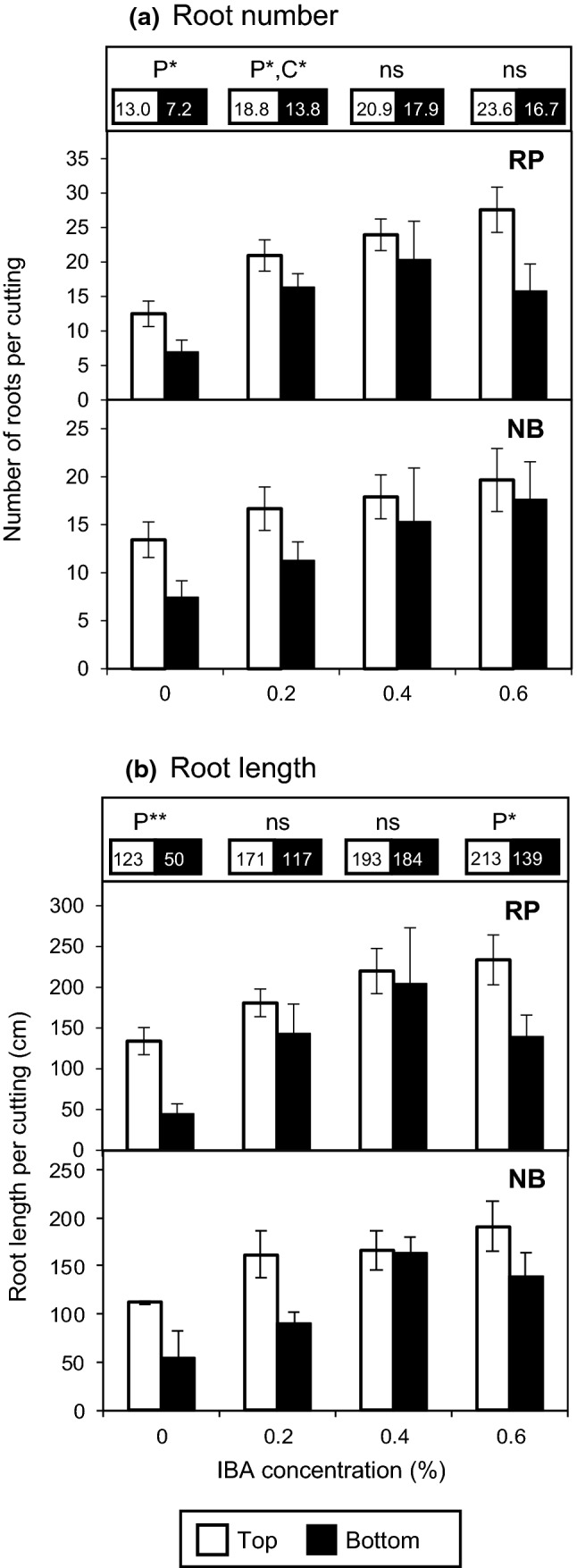
Number (**a**) and total length (**b**) of adventitious roots formed by cuttings of ‘Rosa progress’ (RP) and ‘Natal briar’ (NB) in dependence of cutting position (top, bottom) and concentration of applied indole-3-butyric acid (IBA). Columns and bars represent mean values and SE per cultivar (*n* = 3, each with 20 cuttings). Numbers in the upper parts represent the mean root number (**a**) and mean root length in cm (**b**) of the two cultivars formed by the top cuttings (black letters) versus the bottom cuttings (white letters, black shade). P and C indicate significant effects of position and cultivar, respectively, at the specified level of IBA supply. *, **Significant at *P* ≤ 0.05, 0.01, respectively; ns, neither cultivar nor position was significant

Since the analysis of IAA and CKs uncovered auxin as the main hormone class responding to the nodal position of rose cuttings further determining the auxin/cytokinin balance, we investigated the response of AR formation in the cuttings to the nodal position and to external auxin supply (Fig. 7). Without auxin application, bottom cuttings of both cultivars produced significantly lower numbers (Fig. 7a) and lengths of ARs (Fig. 7b) than top cuttings. When the auxin indole-butyric acid (IBA) was supplied to the cuttings, increasing concentrations up to 0.4% enhanced the number and length of ARs in top and bottom cuttings of both cultivars. However, this treatment enhanced AR formation more in bottom than in top cuttings, so that independent on cultivar at 0.4% IBA cuttings from both positions produced the same number (Fig. 7a) and length (Fig. 7b) of ARs. Higher supply of IBA did not further increase rooting but rather decreased ARs formation in bottom cuttings, which at 0.6% IBA revealed a decreased root length when compared with top cuttings. As whole, these results document a deficit in rooting capacity of bottom cuttings of both cultivars, when they rely on their endogenous auxin. However, this deficit can be counterbalanced by optimum auxin supply, while the bottom cuttings obviously also reveal a higher sensitivity to excessive auxin than top cuttings.

## Discussion

We investigated the relationship between auxin, cytokinins and adventitious root formation of leafy single-node stem cuttings of rose under the influence of different nodal positions within the shoots of the stock plant. To detect interferences with plant genotype, two cultivars were compared. One important characteristic of nodal stem cuttings is that excision from the stock plant involves wounding not only in the stem base region but also at the apical end of the cutting and, most important, causes the release from apical dominance. The cutting and particularly the axillary bud are isolated from the apical part of the shoot, which constitutes an important source of auxin in the intact plant, so that the outgrowth of buds is initiated. To date, the regulative communication between bud outgrowth and AR formation is not understood (Bredmose et al. 2005). To account for this complexity, we investigated the auxin and CK concentrations in the stem base and in the bud region, which included the node and the bud in the leaf axis (Fig. 1).

### Higher nodal position of cuttings stimulates auxin accumulation in the stem base, while outgrowing buds seem to function as auxin source

IAA concentrations increased in the bud region after excision and planting of cuttings, while independently on cultivar top cuttings accumulated significantly higher IAA levels until 8 DAP than bottom cuttings (Figs. 3c and 4a, b). Cuttings sampled at 4 DAP and 8 DAP already showed bursted and elongated buds, respectively, while top cuttings showed advanced outgrowth of buds (Table 2). In another study, excised rose buds accumulated IAA during in vitro cultivation for 100 h in dependence on sucrose that stimulated the outgrowth of buds (Barbier et al. 2015). The increase in IAA levels corresponded to enhanced expression of the *RhTAR1* and *RhYUC1* genes, that control IAA biosynthesis via the tryptophan-dependent YUCCA pathway (Barbier et al. 2015). In Arabidopsis leaf explants, expression of two YUCCA genes was up-regulated after excision of the leaves, whereas genetic and pharmacological inhibition of the YUCCA pathway inhibited AR formation in the explants (Chen et al. 2016). Considering these findings, the temporal increase and higher IAA accumulation in the bud region of top cuttings probably result from stimulation of local IAA biosynthesis in the buds. However, also other processes that control auxin homeostasis in cutting tissues such as reduced conjugation or catalysis of IAA or enhanced release of IAA from conjugates may be involved (Druege et al. 2014).

The transient decrease of IAA levels in the stem base between 0 and 1 DAP was not statistically significant but appeared as consistent for the four combinations of cultivar and cutting position (Fig. 4c, d). This may be the consequence of dis-coupling from the apical auxin source of the shoot and a lag time, before auxin biosynthesis was up-regulated in the buds, but also influenced by local metabolic processes such as auxin conjugation. In the stem base of petunia shoot tip cuttings, genes of the *GH3* gene family, that encode acyl acid amido synthetases some of which may conjugate IAA to amino acids, were strongly upregulated within few hours after cutting excision (Druege et al. 2014). After the transient decrease, IAA concentrations increased in the stem base to significantly higher levels in the top cuttings (Fig. 4c, d). The corresponding dynamic of IAA in the bud region and in the stem base and the highly significant positive correlations of IAA levels between both cutting parts at 8 DAP (Fig. 5a) support the view that the temporal increase in IAA in the stem base and the enhanced levels in top cuttings at 8 DAP are the consequence of enhanced IAA influx from the bud region. The important functions of polar auxin transport in cutting stems, of PIN1 as important auxin efflux transporter and of the upper cutting parts as auxin source for local auxin accumulation in the stem base is supported by findings on shoot tip cuttings of carnation (Garrido et al. 2002) and petunia (Ahkami et al. 2013; Yang et al. 2019). Furthermore, in vitro cultivated excised rose buds were shown to express *RhPIN1* and *RhPP2A* that putatively control auxin efflux and basal localization of PINs in cells, in a sucrose-dependent manner (Barbier et al. 2015). In the same study, the contribution of axillary buds to the auxin homeostasis of stems was supported by experiments with a DR5::GUS expressing pea line. GUS staining in leafless bud-bearing stem sections revealed an intense auxin cannel between the bud and the stem, starting at 48 h after excision of the stem section, when sucrose was supplied (Barbier et al. 2015). It can be expected that after planting of cuttings and cultivation under light in the present study, the fully developed leaf adjacent to the bud (Fig. 1) provided the sucrose to stimulate auxin biosynthesis and export from the bud. Interestingly in this context, studies of nutritional factors in rose cuttings of the same cultivars as in the present study revealed, that top cuttings contained higher sucrose levels than bottom cuttings (Otiende et al. 2017).

The higher IAA levels in the stem base of top cuttings of the cultivar ‘Natal Briar’ at 0 DAP and of the cultivar ‘Rosa progress’ at 2 DAP (Fig. 4c, d), that contributed to the significant higher IAA levels in top cuttings over the first week (Fig. 2a) do not appear as related to an enhanced auxin source in the buds (Fig. 4a, b). Other processes, which may have been manifested at stock plant level already, may be involved. This may include enhanced polar auxin transport to the stem base, decreased catalysis of IAA, e.g., via lower peroxidase activity, higher local IAA biosynthesis or less conjugation of IAA to sugars and amino acids (Osterc and Stampar 2011; Osterc et al. 2016; Woodward and Bartel 2005). Zinc is a well-known factor that is important for the biosynthesis of tryptophan, a precursor of IAA (Cakmak et al. 1989). Most interestingly, under same experimental conditions as of the present study, top cuttings of ‘Rosa progress’ and ‘Natal briar’ revealed significantly higher zinc concentrations in the stem base than bottom cuttings at time of planting, while highest levels were found in ‘Natal briar’ (Otiende et al. 2017).

### Release from apical dominance with cutting excision causes an early cytokinin accumulation that triggers auxin biosynthesis in the bud

In our study, we focused on the CKs of the IP- and zeatin-type in the tissues of *R. hybrida* ‘Natal Briar’ and ‘Rosa Progress’ and detected tZ, tZR, cZR, DHZR and IPR. Roman et al. (2016) additionally detected IP in buds and nodes of *R. hybrida* ‘Radrazz’, but maximum concentrations reached only 3% of the maximum level of IPR. Probably, our method was not sensitive enough for the detection of the low IP levels. We observed a rise in levels of most CKs during the first two days after planting of the cuttings, which was most pronounced in the bud region. This stays in contrast to the mostly observed decrease of CK levels in shoot tip cuttings (Druege et al. 2019) and can be explained by stimulation of CK biosynthesis in the cutting tissues after release from apical dominance. It has been shown in *Pisum sativum* and Arabidopsis that with decapitation of plants the IAA repression of the expression of the *isopentenyl transferase (IPT)* genes that control cytokinin biosynthesis in the node terminates and this allows for the increased cytokinin levels in the same organ (Tanaka et al. 2006; Müller et al. 2015). In the present study, CKs accumulated in the bud region of rose until 2 DAP (Figs. 4e, f, i, j and 6a, b), which was 2 days before the first indication of starting outgrowth of buds (Table 2). Similarly, Bredmose et al. (2005) found an increase in CK levels in the bud region of single-node cuttings of *Rosa* L. “Poulra002^N^” Heidi until the onset of bud outgrowth. Roman et al. (2016) investigated the regulation of genes controlling CK and auxin homeostasis in response to decapitation of plants of *R. hybrida* ‘Radrazz’. They showed that genes controlling CK biosynthesis such as *RhIPT3 and RhIPT5* and of genes that control CK activation (converting of nucleotide forms into active forms) and a putative CK transporter are up-regulated in the node soon after decapitation causing CK accumulation in the bud in dependence on white light. Inhibitors of CK biosynthesis or perception inhibited bud outgrowth, whereas CK application including IP could substitute light in stimulation of decapitation-induced bud outgrowth and in upregulation of the *PhYUC1* and *RhPIN1* genes that control auxin biosynthesis and transport in rose (Roman et al. 2016). In the light of these findings, the data of the present study strongly support the conclusion that after excision of the cuttings, light-dependent stimulation of CK biosynthesis in the node in response to the release from apical dominance stimulated biosynthesis of auxin in the outgrowing buds, that was then basipetally transported to stem base. Considering the substantial, up to tenfold relative increase of IPR particularly in the bud region until day 2 (Fig. 4e, f) and the basipetal phloem transport of this CK (Kudo et al. 2010; Skalicky et al. 2018), bud-derived IPR may have particularly supported the observed increase of CKs in the stem base (Fig. 4g, h).

### Higher IPR levels in top position cuttings may push auxin biosynthesis

The higher concentrations of IPR and the increased ratios of IPR to Z-type CKs found in the chronologically younger tissue of the top cuttings (Figs. 2d, e and 4e–h) correspond to effects of plant age and development on CKs reported for rose and other woody plant species. In leaves of the main shoot and in axillary shoots of *R. hybrida*’ Madelon’ grafted ‘on ‘Maltic’, levels of IPR and the ratios of IP-type to Z-type CKs decreased chronologically, when plants developed from the stage of primary shoot bearing two short axillary shoots to the stage of flowering of the elongated axillary shoots (Dieleman et al. 1997). In *Pinus radiata, P. pinea* and *Prunus persica*, levels of IP and/or IPR and IPR/Z-type CKs ratios were higher in needles, terminal buds or axillary buds of young, juvenile plants when compared with older, more mature trees (Moncalean et al. 2002; Valdes et al. 2002, 2004a). In *P. radiata* trees of same age, levels of IP and IPR decreased with increasing chronological age of the needles (Valdes et al. 2004b). The specific functions of IP-type CKs in relation to plant age and maturation are not understood. Interestingly, in the present study, top position cuttings of both cultivars accumulated significantly more IPR until 4 DAP in the cutting tissues when compared with bottom cuttings (Fig. 4e–h). Further, the higher IAA levels that were attained in the bud region of top cuttings at 8 DAP were positively correlated to previous IPR levels in same tissues (Fig. 5b). Even though the regression lines were cultivar-specific, considering the reported light-dependent early accumulation of IPR in the node of *R. hybrida *‘*Radrazz*’, obviously triggering the decapitation-induced stimulation of auxin biosynthesis and transport in the buds (Roman et al. 2016), the present results strongly suggest that the higher IPR levels in the top cuttings contributed to the enhanced auxin levels and the advanced outgrowth of buds in the cuttings.

### Enhanced auxin levels and higher auxin/CK ratio in top position cuttings promote induction of ARs

Recently, it was shown that the diversity of number of ARs formed ex vitro by leafy nodal cuttings of 96 rose genotypes without auxin application was associated with SNPs located in genes that putatively control auxin signaling such as auxin response factors, *Scarecrow* and *Wuschel-related homebox 8-*like genes, whereas numbers of ARs formed in vitro under external auxin supply showed only weak relations to such genes (Nguyen et al. 2020). This finding already documents the important role of auxin homeostasis in rose cuttings in limiting AR formation upstream of the auxin signal transduction chain. In the present study, higher IAA levels were found in the stem base of top versus bottom cuttings between 0 and 8 DAP (Figs. 2a and 4c, d) and these further determined a higher auxin/CK ratio in same tissues (Fig. 5c, d). To provide evidence for a functional role of auxin in the acropetal increase in rooting capacity of rose, we tested whether the impaired rooting of bottom cuttings can be rescued by auxin application. We used the natural auxin indole-butyric acid (IBA) because of its known stable positive effects on AR formation in cuttings compared to other available auxins (Lakehal and Bellini 2019) and because in Arabidopsis the function of IBA has mainly been attributed to its conversion to IAA (Müller 2020). Our finding, that basal IBA application at a concentration of 0.4% counterbalanced the deficit in rooting of bottom cuttings (Fig. 7), strongly support the conclusion that the higher IAA level in top cuttings is the crucial factor contributing to the improved rooting.

Information about the timing of the successive phases of AR formation in rose cuttings is fragmentary. In our study, no ARs were visible until 8 DAP, the last date of hormone analysis, when first callusing was detected only in top cuttings of ‘Rosa progress’ (Table 2). In single-node cuttings of *Rosa helenae* ‘Semiplena’, external callus was visible at ca. 7 days after excision and first roots were visible between 21 and 28 days, while anatomical studies at day 25 discovered root primordia inside the stem, that obviously originated from the cambium and phloem region (Monder et al. 2019). In light of these findings and considering the temporal distribution of IAA in the present study, it can be expected that AR induction occurred during the period until 8 DAP, when the top cuttings revealed an auxin–CK homeostasis being more supportive for AR induction than the bottom cuttings. Whether the higher IPR levels in the stem base of top cuttings (Fig. 4g, h) may have additionally supported early CK-dependent dedifferentiation of AR source cells (Druege et al. 2019), requires further investigations.

## Conclusion

This study has provided evidence that higher position of nodal rose cuttings within the donor shoot involves higher CK levels of the IP-type and higher IAA levels in the bud region and the stem base, while the higher IAA level further determines a higher IAA/CK ratio in the stem base during the first week after cutting excision. The findings, that the acropetal increase of IAA levels in the bud region and in the stem base and the resulting higher IAA to CK ratio was consistent between the two cultivars and that independent on cultivar the deficit in AR formation of bottom versus top position cuttings was counterbalanced by external auxin supply, highlights the functional role of auxin in the acropetal stimulation of AR formation and support the conclusion that the found relationships are highly relevant to the frequently observed better rooting of apical cuttings of *R. hybrida*. The dynamic of auxin and CK levels in the two cutting parts and of the outgrowth of buds support the view that a CK-stimulated auxin biosynthesis in the buds contributes to the auxin homeostasis in the stem base, which is obviously pushed in top cuttings by the higher levels of phloem-mobile IPR.

**Fig. 8:**
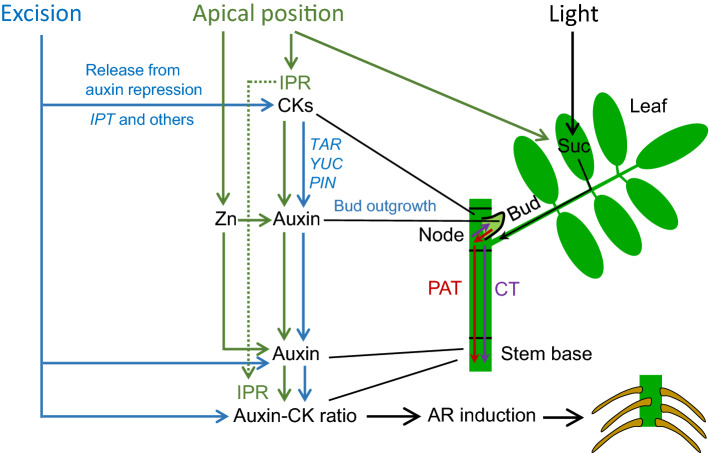
Working model of auxin–cytokinin interactions during AR formation in leafy nodal cuttings of rose as affected by excision of cuttings and nodal position within the donor shoot, based on the presented results and earlier studies of AR formation and axillary bud growth in rose (Barbier et al. 2015; Roman et al. 2016; Otiende et al. 2017). Excision of cuttings causes wounding and isolation from the apical and basal shoot. The release from the apical dominance enhances CK biosynthesis in the node, involving the release of *ISOPENTENYLTRANSFERASE* (*IPT*) genes from auxin repression. CKs are transported into the bud, where they stimulate their outgrowth and auxin biosynthesis and polar auxin transport (PAT) via upregulation of *TRYPTOPHAN AMINOTRANSFERASE RELATED* (*TAR*), *YUCCA* (*YUC*) and *PIN-FORMED* (*PIN*)*1*. Light powers the supply with sucrose as trigger of auxin biosynthesis and transport in the bud. The IAA is transported via PAT to the stem base, where the enhanced IAA level and enhanced auxin/CK ratio initiates the AR induction. Cuttings from more apical position accumulate higher levels of IPR and reach higher IAA levels and IAA/CK ratios particularly in the stem base during the first week after planting. This is initiated by the stimulation of CK-mediated auxin biosynthesis in the buds, while phloem-mobile IPR has a particular function. Auxin biosynthesis is further stimulated by the higher content of zinc (Zn) as important factor of tryptophan biosynthesis and by the higher sucrose levels that stimulate expressions of *TAR*, *YUC* and *PIN1* in the bud. The resulting higher IAA levels and higher IAA/CK ratio in the stem base of apical cuttings stimulate AR induction. Blue and green arrows indicate promotive influences of cutting excision and apical position of cuttings, respectively. Red and violet arrows represent the major directions of auxin flow by PAT and of CK transport (CT) via IPR in the phloem, respectively

In light of the presented findings, of our preliminary findings concerning nutritional factors in same cuttings (Otiende et al. 2017) and of reported effects of decapitation on axillary bud growth (Barbier et al. 2015; Roman et al. 2016) a working model of auxin–CK interactions in excision-induced and nodal position-mediated AR formation in rose is presented in Fig. 8. Excision of cuttings involves the release from apical dominance, causing a decrease of IAA in the node. This enhances biosynthesis of CKs, which are transported into the bud, where they stimulate their outgrowth, auxin biosynthesis and polar auxin transport (PAT). Light powers the supply with sucrose as trigger of auxin biosynthesis and transport. The IAA is transported via PAT to the stem base, where the enhanced IAA level determines an enhanced auxin/CK ratio initiating the AR induction. Accumulation of IAA may be supported by wound-induced local biosynthesis or release from conjugates and by the cut-off from the basipetal auxin drain. When cuttings are excised from more apical positions, they contain higher levels of IPR and reach higher IAA levels and IAA/CK ratios particularly in the stem base during the first week after planting. This is initiated by the stimulation of CK-mediated auxin biosynthesis in the buds, while the higher levels of phloem-mobile IPR has a particular function. Auxin biosynthesis in the cutting is further stimulated by the higher content of Zn as important factor of tryptophan biosynthesis and by the higher sucrose levels. The resulting higher IAA levels and higher IAA/CK ratio in the stem base of apical cuttings stimulate AR induction. In addition to these processes, the higher levels of IPR in the apical cuttings may favor early dedifferentiation. Further details and putatively involved genes controlling auxin and cytokinin homeostasis and transport are explained in the figure.

Further work should target the putative processes at organ and tissue level and functionally analyze the contribution of genes that control auxin and CK homeostasis and signaling in nodal cuttings.

### *Author contribution statement*

MAO conducted the experiments, extracted the samples for hormone analysis, analyzed the data and wrote a first draft of the article. KF performed the auxin analysis. MRH performed the cytokinin analysis. JON and KN co-supervised the study. UD conceived and supervised the study, analyzed the data, developed the working model and wrote the article. All the authors edited and approved the manuscript.

## Supplementary Information

Below is the link to the electronic supplementary material.

Supplementary file1 (PDF 269 KB)

## Data Availability

All data generated or analyzed during this study are included in this published article and its supplementary information files.

## Abbreviations

AR: Adventitious root
CK: Cytokinin
DAP: Days after planting
DHZR: Dihydrozeatin riboside
IP: Isopentenyladenine
IPR: Isopentenyladenosine
cZR: *cis*-Zeatin riboside
tZ: *trans*-Zeatin
tZR: *trans*-Zeatin riboside
