# Supplementary material for: Involvement of the auxin–cytokinin homeostasis in adventitious root formation of rose cuttings as affected by their nodal position in the stock plant
Source: Planta. 2021 Sep 6;254(4):65. doi: 10.1007/s00425-021-03709-x (PMC8421306; doi:10.1007/s00425-021-03709-x)
Supplement: Supplementary file 1 — Supplementary file1 (PDF 269 KB) [file 425_2021_3709_MOESM1_ESM.pdf]

Supplementary information (SI) 1

to the article entitled

Involvement of the auxin-cytokinin homeostasis in adventitious root formation of rose cuttings as affected by their nodal position in the stock plant

published in
Planta

Authors:
Millicent A. Otiende, Klaus Fricke, Julius O. Nyabundi, Kamau Ngamau, Mohammad R. Hajirezaei, Uwe Druege

For correspondence:
Uwe Druege, Erfurt Research Centre for Horticultural Crops, University of Applied Sciences Erfurt, 99090 Erfurt, Germany
e-mail: uwe.druege@fh-erfurt.de

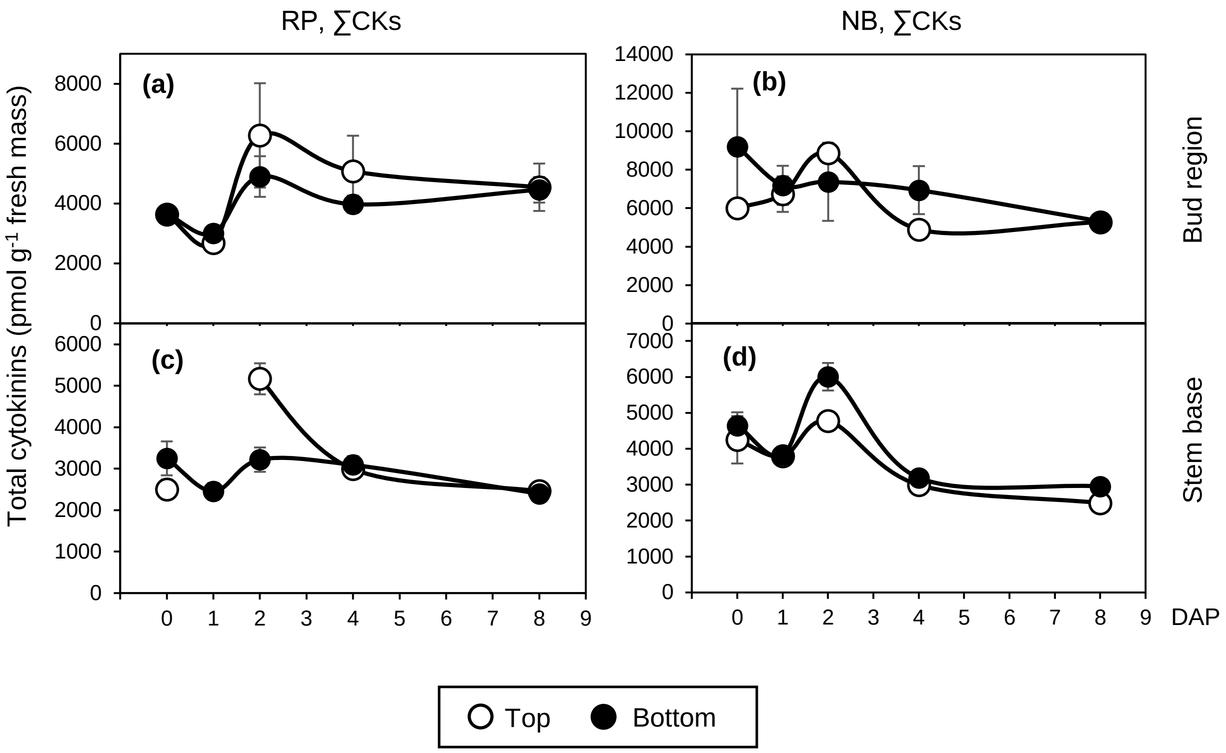

Supplemental Figure S1 Concentration of total cytokinins (∑CKs in the bud region (**a**, **b)** and the stem base (**c, d**) of cuttings of the *Rosa hybrida* cultivars ‘Rosa progress‘ (RP, **a, c**) and ‘Natal briar‘ (NB, **b, d**) as affected by harvest position of cuttings within the stock plant (top, bottom) and days after planting (DAP). Mean values and SE (n = 4)

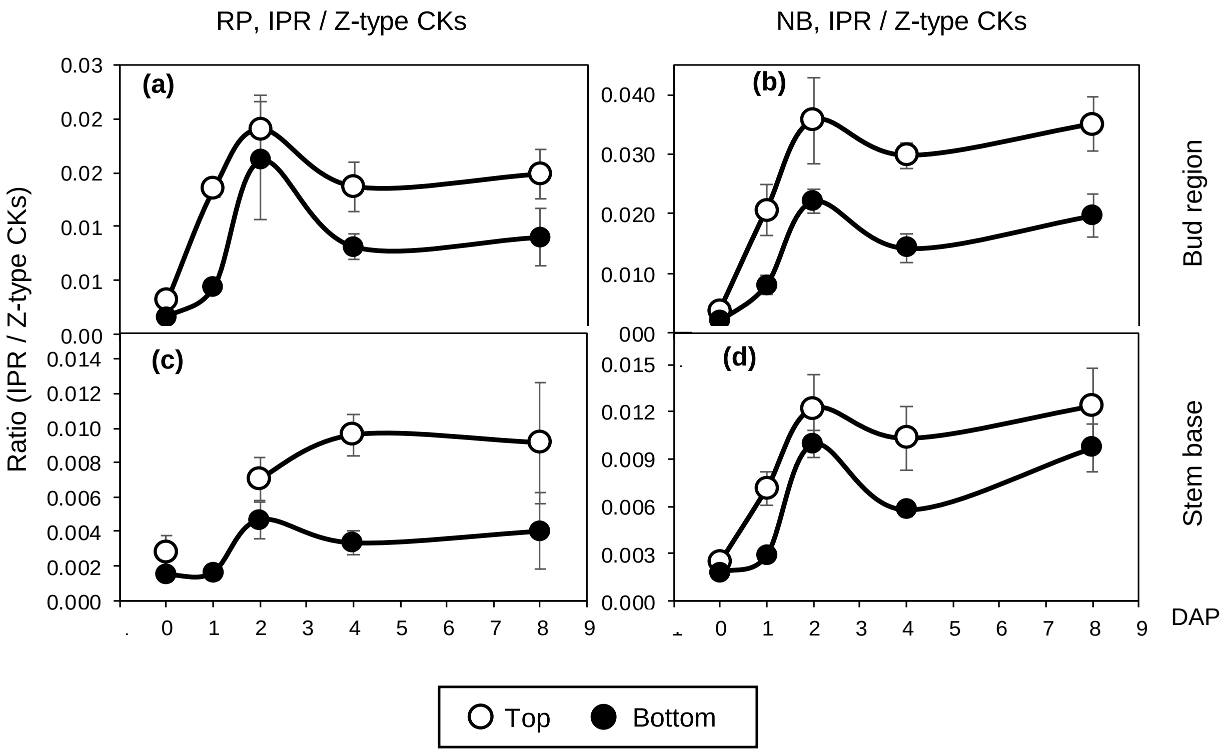

Supplemental Figure S2: Ratio of IPR to Z-type cytokinins (CKs) in the bud region (**a, b**) and the stem base (**c, d**) of cuttings of the *Rosa hybrida* cultivars ‘Rosa progress‘ (RP, **a, c**) and ‘Natal briar‘ (NB, **b, d**) as affected by harvest position of cuttings within the stock plant (top, bottom) and days after planting (DAP). Mean values and SE (n = 4)

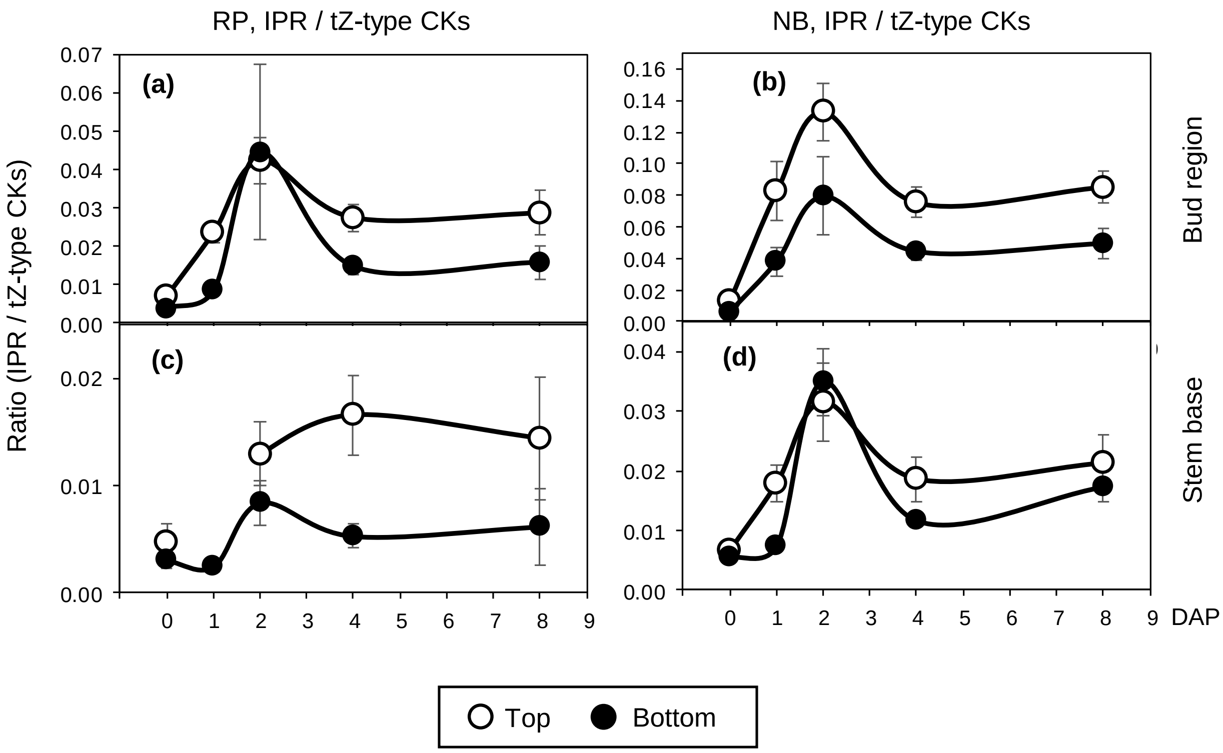

Supplemental Figure S3: Ratio of IPR to trans-Z-type cytokinins (CKs) in the bud region (**a**, **b)** and the stem base (**c, d**) of cuttings of the *Rosa hybrida* cultivars ‘Rosa progress‘ (RP, **a, c**) and ‘Natal briar‘ (NB, **b, d**) as affected by harvest position of cuttings within the stock plant (top, bottom) and days after planting (DAP). Mean values and SE (n = 4)

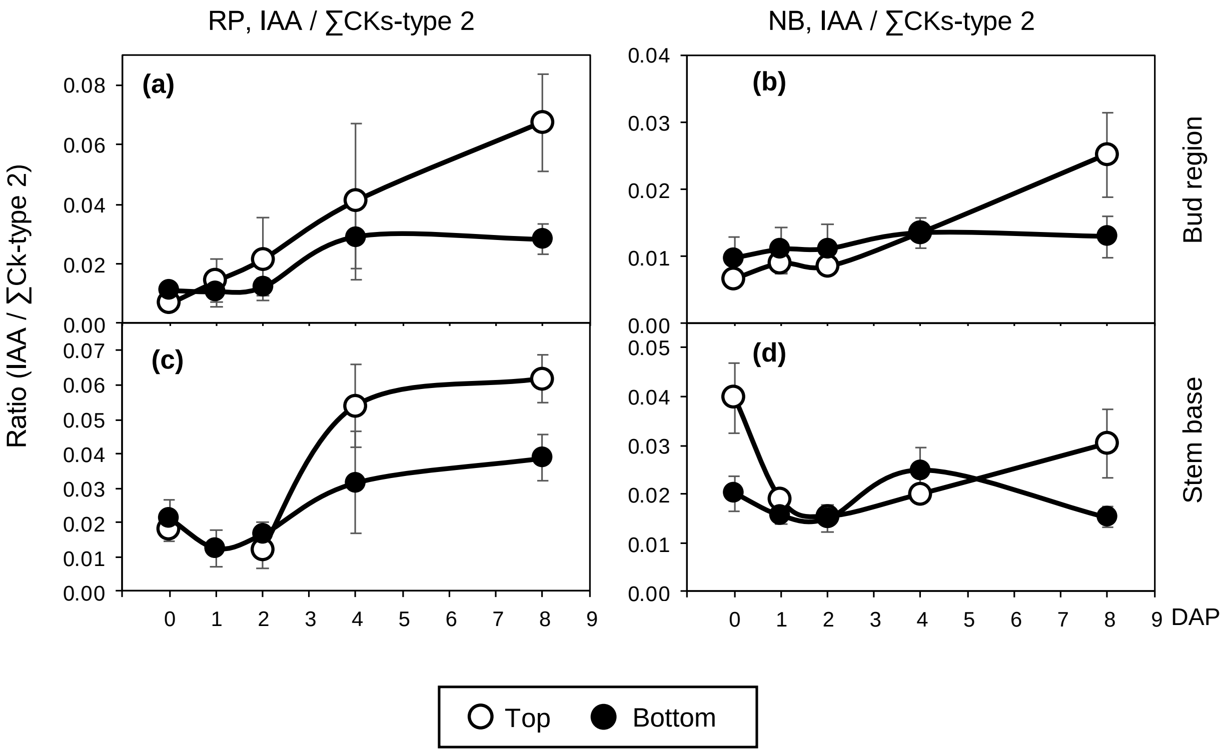

Supplemental Figure S4: Ratio of IAA to total cytokinins (∑CKs) of type 2 (excluding cis ZR) in the bud region (**a, b**) and the stem base (**c, d**) of cuttings of the *Rosa hybrida* cultivars 'Rosa progress' (RP, **a, c**) and 'Natal briar' (NB, **b, d**) as affected by harvest position of cuttings within the stock plant (top, bottom) and days after planting (DAP). Mean values and SE (*n* = 4)
